# Highly Loaded Cellulose/Poly (butylene succinate) Sustainable Composites for Woody-Like Advanced Materials Application

**DOI:** 10.3390/molecules25010121

**Published:** 2019-12-28

**Authors:** Oskars Platnieks, Sergejs Gaidukovs, Anda Barkane, Gerda Gaidukova, Liga Grase, Vijay Kumar Thakur, Inese Filipova, Velta Fridrihsone, Marite Skute, Marianna Laka

**Affiliations:** 1 Faculty of Material Science and Applied Chemistry, Institute of Polymer Materials, Riga Technical University, P.Valdena 3/7, LV, 1048 Riga, Latvia; oplatnieks@gmail.com (O.P.); barkaneanda@gmail.com (A.B.); 2 Faculty of Material Science and Applied Chemistry, Institute of Applied Chemistry, Riga Technical University, P.Valdena 3/7, LV, 1048 Riga, Latvia; Gerda.Gaidukova@rtu.lv; 3 Faculty of Material Science and Applied Chemistry, Institute of Silicate Materials, Riga Technical University, P.Valdena 3/7, LV, 1048 Riga, Latvia; grase.liga@gmail.com; 4 School of Aerospace, Transport, and Manufacturing, Cranfield University, Cranfield, Bedfordshire MK43 0AL, UK; vijay.kumar@cranfield.ac.uk; 5 Latvian State Institute of Wood Chemistry, LV, 1006 Riga, Latvia; inese.filipova@inbox.lv (I.F.); fridrihsone.velta@inbox.lv (V.F.); polarlapsa@inbox.lv (M.S.); lamar@edi.lv (M.L.)

**Keywords:** cellulose, poly (butylene succinate) composite, physical-mechanical properties, thermo-mechanical properties, sustainable woody-like composites

## Abstract

We report the manufacturing and characterization of poly (butylene succinate) (PBS) and micro cellulose (MCC) woody-like composites. These composites can be applied as a sustainable woody-like composite alternative to conventional fossil polymer-based wood-plastic composites (WPC). The PBS/MCC composites were prepared by using a melt blending of 70 wt% of MCC processed from bleached softwood. MCC was modified to enhance dispersion and compatibility by way of carbodiimide (CDI), polyhydroxy amides (PHA), alkyl ester (EST), (3-Aminopropyl) trimethoxysilane (APTMS), maleic acid anhydride (MAH), and polymeric diphenylmethane diisocyanate (PMDI). The addition of filler into PBS led to a 4.5-fold improvement of Young’s modulus E for the MCC composite, in comparison to neat PBS. The 1.6-fold increase of E was obtained for CDI modified composition in comparison to the unmodified MCC composite. At room temperature, the storage modulus E′ was found to improve by almost 4-fold for the APTMS composite. The EST composite showed a pronounced enhancement in viscoelasticity properties due to the introduction of flexible long alkyl chains in comparison to other compositions. The glass transition temperature was directly affected by the composition and its value was −15 °C for PBS, −30 °C for EST, and −10 °C for MAH composites. FTIR indicated the generation of strong bonding between the polymer and cellulose components in the composite. Scanning electron microscopy analysis evidenced the agglomeration of the MCC in the PBS/MCC composites. PMDI, APTMS, and CDI composites were characterized by the uniform dispersion of MCC particles and a decrease of polymer crystallinity. MCC chemical modification induced the enhancement of the thermal stability of MCC composites.

## 1. Introduction

In recent years, the global trends have been changing and renewable materials and resources have become an important part of our life. One of the main concerns for polymer-based materials is their carbon contribution and growing environmental pollution [1]. Bio-based polymer monomers have partly solved these problems, but only a few polymers meet the criteria of biodegradation. Polyesters are widely considered as a replacement for polyolefins due to their meeting sustainability goals. Polybutylene succinate (PBS) is polyester that can be produced fully or partly from bio-based monomers and is biodegradable [2,3]. PBS properties have been compared to polyolefins, but bio-based polymer prices are several times higher than oil-based ones and provide economic challenges [4,5].

The preparation of cellulose composites can overcome inherited polymer matrix issues [6]. Cellulose-based biomass has become one of the most used filler materials for composite preparation. It is a relatively cheap material, is often discarded as waste, and has residues from forestry and agricultural industries. The fiber structure of cellulose offers low density and high specific strength, stiffness, and is non-toxic to humans and nature [7]. Different sources of cellulose fillers are used for polymer composite fabrication [8], e.g., lignocellulosic fibers [9], microcrystalline cellulose [10], and nanocellulose [11]. Abundance, high strength, stiffness, low weight, and the biodegradability of the micro-fibrillated cellulose (MCC) underscores it as a very promising candidate for polymer composite preparation [12].

MCC is obtained from wood pulp treated with acid [10]; this results in cellulose microparticles with high crystallinity and a large surface area compared with other cellulose fibers [13]. Studies of MCC modified polymer composites with various matrixes like polyethylene [8], polypropylene, nylon, polyethylene terephthalate, polyurethane, and many others have been performed [14,15,16]. General studies with MCC filler report increased mechanical and/or dynamic mechanical properties, but properties generally decrease above 30 wt% filler loading. Cellulose molecules have several hydroxyl groups, which form hydrogen-bond networks and are responsible for high strength and the stiffness of cellulose fibers, but unfortunately, the polar nature of cellulose shows weak interactions with a non-polar matrix of polymer, resulting in agglomerations that limit possible composite improvements [17].

Cellulose modification methods have been used successfully to further improve mechanical properties, compared to non-modified cellulose composites [18]. Treatment with organosilanes is one of the most used modification methods for coupling material with polymers [19]. Silane coats the surface of the polar material with hydroxyl groups and permanent bonds can be obtained. There is often no chemical reaction between silane and polymer, but silane contains alkyl groups that increase the compatibility with non-polar matrix. Maleic anhydride (MAH) has also been used as a grafting agent to promote the reaction between cellulose-based fillers [20]. MAH provides a significant increase in mechanical properties and is suitable for the large scale production of composites [21]. Treatment with isocyanates is reported in scientific publications, while being very widely used in the industry, mainly for polyurethane composite material production [22]. The cellulose surface can easily be chemically treated with isocyanates during processing, while the chain reaction with isocyanate can develop a fully cross-linked system [23]. Cellulose coating with alkyl ester and hydroxy amide surfactants seem to be one of the easiest and safest modification methods that we find particularly interesting and different from other approaches [24,25]. These modification methods to improve the interfacial interactions through compatibilization are well known in the literature. They are very effective in reducing the impact of the negative effects of poor interfacial adhesion and even further improve the performance of the polymer composites [26].

Considering the significant impact of MCC chemical modification on the PBS/MCC composite properties and structure, six different chemical treatment strategies have been investigated in this paper, with a focus on understanding the most suitable chemical modification method to complement the chosen polymer and filler. PBS composites with high MCC loading of 70 wt% were melt processed, while carbodiimide (CDI), polyhydroxy amides (PHA), alkyl ester (EST), (3-Aminopropyl) trimethoxysilane (APTMS), maleic acid anhydride (MAH), and polymeric diphenylmethane diisocyanate (PMDI) treatments were carried out for the manufacturing of sustainable woody-like PBS/MCC composites. The set objectives were to characterize the thermal, physical-mechanical properties, thermo-mechanical, and structural properties of sustainable woody-like PBS/MCC composites.

## 2. Results and Discussion

### 2.1. Thermal Properties

**Figure 1:**
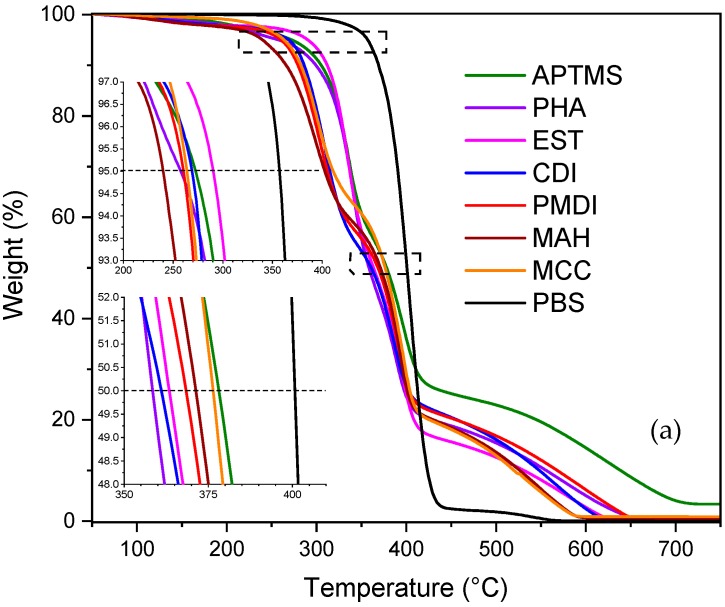

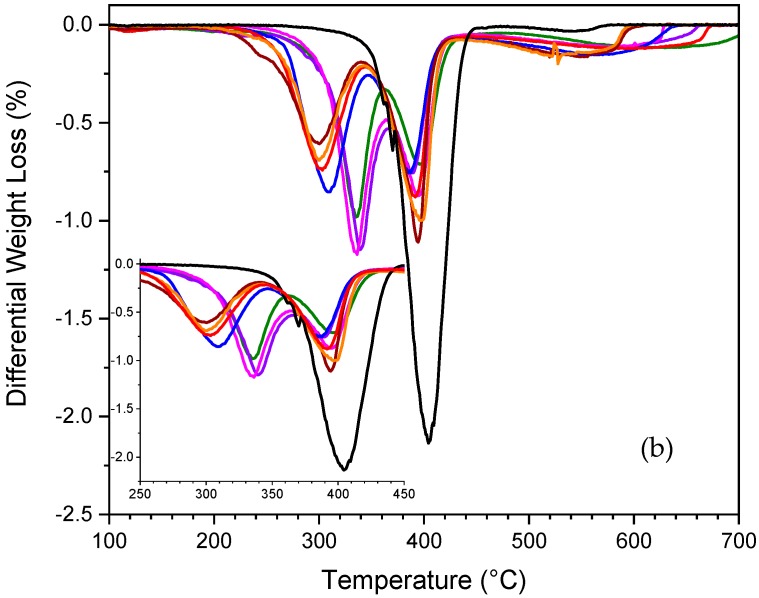
Thermogravimetric analyses curves (**a**) and differential thermogravimetric (**b**) of micro-fibrillated cellulose/ poly (butylene succinate) (PBS/MCC) composites.

**Figure 2:**
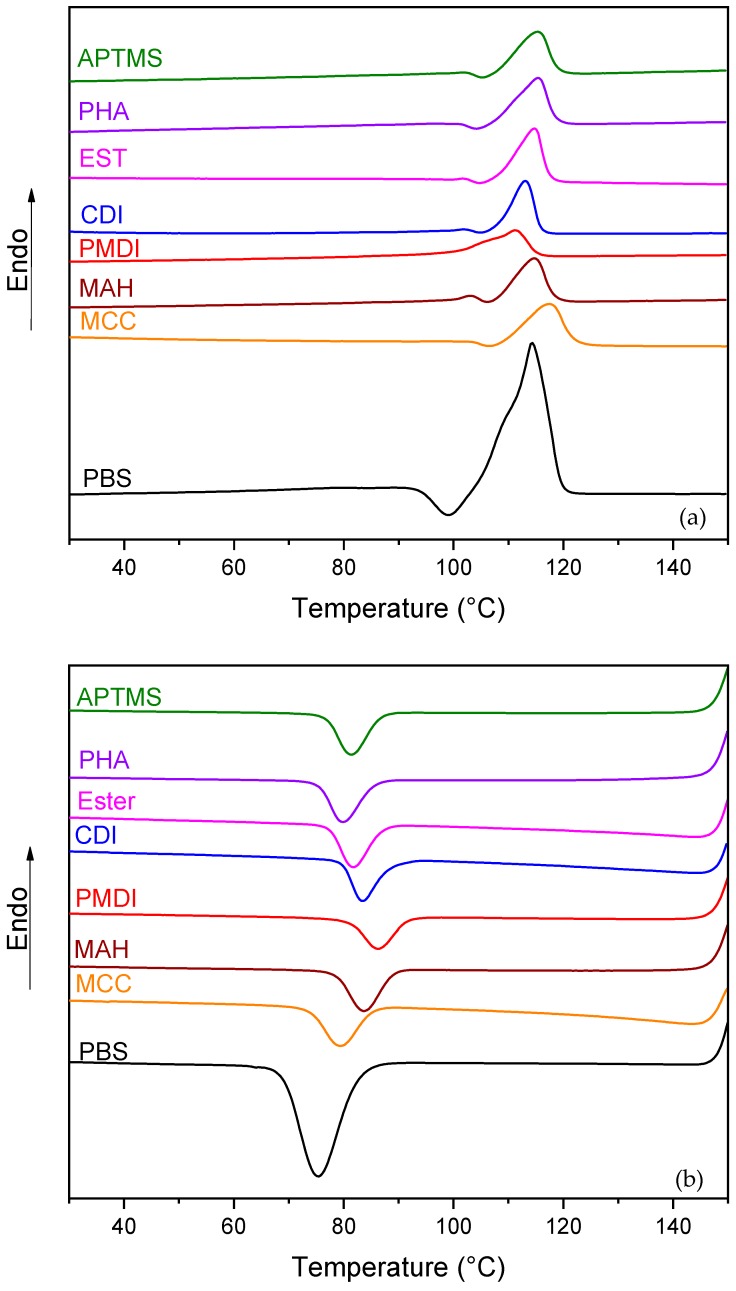
Differential scanning calorimetry curves of PBS/MCC composites: Heating (**a**) and cooling (**b**).

TGA was used to investigate the influence of the composition on the thermal sensitivity and degradation properties of PBS/MCC composite materials. Figure 1 shows the thermogravimetric analyses (TGA) (a) and differential thermogravimetric (DTG) (b) curves of PBS/MCC composites. All PBS/MCC compositions showed a small mass loss of 2% below 100 °C, mainly corresponding to the removal of residual water [27]. The degradation mechanism and degradation temperature of neat PBS and c PBS/MCC composites differed strongly. It is assumed that MCC had a lower thermal stability than neat PBS polymer [28,29]. This meant that the incorporation of the MCC induced less thermal stability of PBS/MCC composites. Lee et al. reported this for PBS/kenaf fiber composites [30]. The temperature at 5% mass loss for MAH composition was 240 °C, and for the EST composition, it was 290 °C. The rest of the composites showed a temperature at 5% mass approximately in this 240–290 °C temperature range. The PBS exhibited only single-stage degradation with a peak at 406 °C, whereas MCC filled composites revealed two degradation peaks in the temperature range of 301–398 °C. This indicated that the single-stage thermal degradation process was defined primarily by the PBS polymer chain degradation, while PBS/MCC composite degradation was affected by cellulose incorporation, mutual interaction between polymer matrix and fillers, and filler surface modification. Roman and Winter evidenced the strict relation of cellulose surface modification and its thermal stability [31]. The temperature at 50% mass loss for PBS was 401 °C. The first stage degradation in DTG (Figure 2b) was around 310 °C for the MCC sample; while for MAH, PMDI, and CDI composites, the thermal degradation shifted to a lower temperature of 315 °C, then for EST, PHA, and APTMS composites, the thermal stability increased up to 340 °C. The composites loaded with chemically coupled and modified MCC revealed enhanced thermal degradation temperatures at 50% mass loss up to 360 °C. This was described as the formation of crosslinked structures with the altered chains and inhibited chain release during the formation of the char in the thermal degradation process [32]. The similar behavior of the more thermally stable PBS composite, in comparison to the neat PBS, was reported by Tang et al. [33] using grafted-nanocellulose as reinforcement.

The results of differential scanning calorimetry (DSC) in the form of the heating and cooling thermal curves of PBS/MCC composites are shown in Figure 2. The thermal curves showed a characteristic endothermic melting transition (Figure 2a) and exothermic crystallization transition (Figure 2b). Meaning changes in the shape of the transitions were observed. However, the crystallization process shifted to the higher temperature range. The influence of the MCC usually resulted in either enhanced crystallization characteristics due to the nucleation effect or the obstruction of polymer molecular chains, which limited the growth of polymer crystals [34]. Enhanced crystallization by means of nucleation and trans-crystallization was reported, mainly for polymer composites with cellulose and other filler contents up to 30 wt% [35]. However, the surface modification of cellulose can strongly hinder polymer chain mobility through physical adsorption and entanglement. This continued in the polymer trans crystallization process on the cellulose, which was also ascribed by the observed, pronounced enhancement of the composite’s mechanical and dynamic mechanical characteristics [36]. Generally, the high loading of MCC reduced crystallinity significantly [37]. This was also the case for composites with extremely high loadings—up to 50–80% of MCC. In Table 1, the experimental values of melt temperature (T_m_), crystallization temperature (T_c_), enthalpy of melting (H_m_), enthalpy of crystallization (H_c_), crystallinity (X), density (ρ), and voids (∆) are presented. All samples with modified MCC showed a very pronounced decrease in X compared to neat PBS and the MCC sample. X of neat PBS has been found to be about 68%. In the case of APTMS, PHA, and EST samples, the obtained X decreased until 52%, 56%, and 58%, respectively. Similar findings have also been reported in the literature [37]. The MCC’s silane treatment improved its dispersion in a polymer matrix, reduced agglomeration, and suppressed the crystalline phase more strongly than the PHA and EST modifications of MCC filler [38]. The chain cross-linking and/or chain extension mechanisms could even further limit polymer chain movements and reduce crystallinity [34]. It was observed that crystallinity decreased by about 20% for compositions MAH, PMDI, and CDI, in comparison to neat PBS. Melting temperature T_m_ and crystallization temperature T_c_ of the obtained samples were modestly decreased and increased correspondingly. This meant that the crystallization of polymer chains with altered flexibility and mobility in the composites interfered with the crystallization process [33,35,37], which started earlier at higher temperatures, and resulted in lower crystallinity—by about 30% of the final composite.

### 2.2. Structure and Morphology Characterization

**Figure 3:**
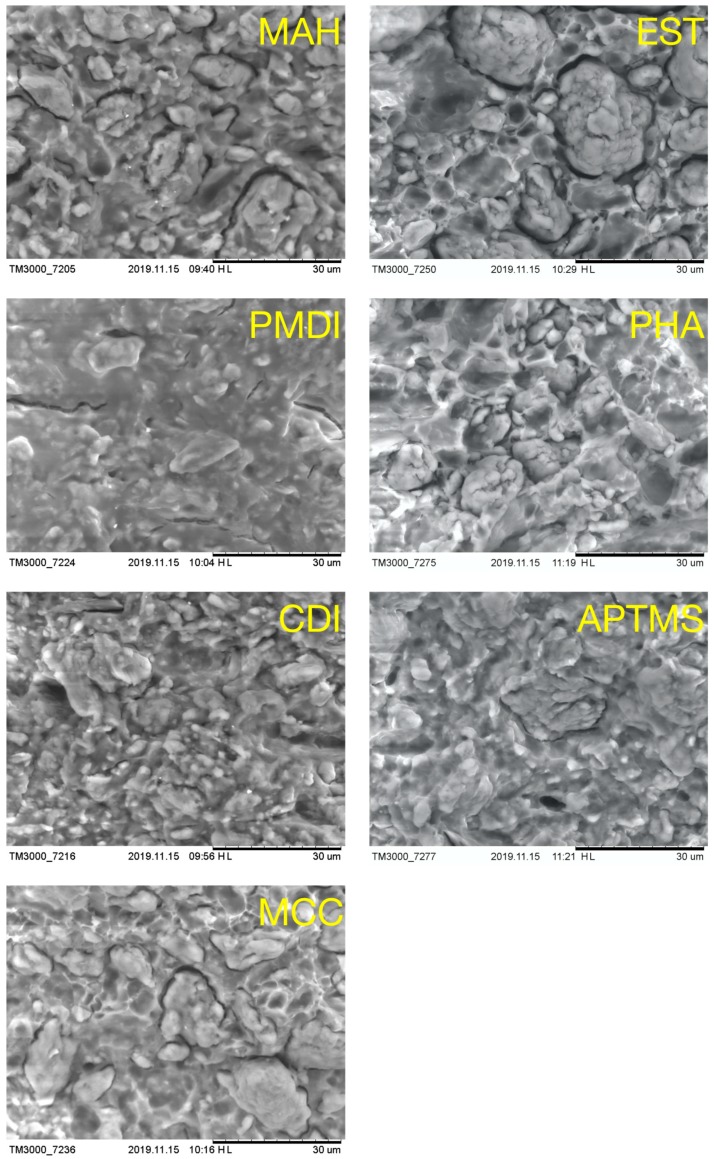
Scanning electron microscopy micrographs of fractured surfaces of PBS/MCC composites.

The experimental density values ρ of the composites, obtained by the weighting method [39], are summarized in Table 1. The value of the parameter ρ *, which is defined as the apparent density of the polymer, was calculated by the equation reported elsewhere [40]. It could correspond to the decrease of the polymer density due to the pronounced drop in crystallinity observed in DSC. SEM images of the fractured surfaces of PBS/MCC composites (Figure 3) evidenced the aggregates of MCC particles. According to the literature [41,42], at high MCC loadings, cellulose particles had a common tendency to aggregate. The fractured surfaces of the MCC sample were very rough. MCC particles could be seen for MCC, MAH, EST, and PHA samples. In turn, CDI, PMDI, and APTMS samples’ surfaces looked smooth, homogenous, and dense in different magnifications (Figure S1). These compositions could be characterized by enhanced compatibility between the polymer matrix and the filler. This was evidenced by the pronounced decrease of crystallinity, dense structure, and uniform dispersion of MCC particles. It also well correlated with the obtained ρ and X values for the PBS/MCC composites. It was reported that the MAH, EST, PHA, CDI, PMDI, and APTMS used could be wetted and dispersed, to some extent, in the cellulose fillers more efficiently in the polymer matrix. MAH could also form an ester linkage between the maleic anhydride and the hydroxyl groups of the cellulose and facilitate the cellulose dispersion [43]. Espino–Pérez et al. established that PMDI is a very effective compatibilizer, as chemical coupling can be established between the isocyanate and the hydroxyl groups on cellulose, and the isocyanate and the carboxylic acid end-groups of the polyester [44]. In turn, the cellulose treatment with the silane was also reported to be the most effective, in comparison to the others, to improve compatibilization and dispersion, which indicated that intramolecular and intermolecular interactions between the cellulose and the polyester were established [45]. 

**Figure 4:**
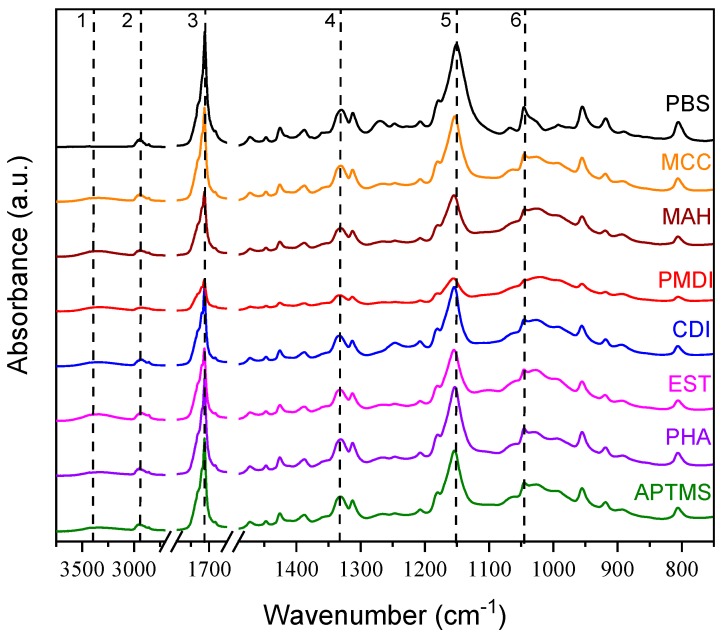
Fourier-transform infrared spectroscopy spectra of PBS/MCC composites.

The characteristic groups of the composites could be evaluated by FTIR spectroscopy. The representative FTIR spectra of the tested compositions are shown in Figure 4 (Figure S2). The characteristic absorption peaks associated with the components were highlighted. The absorption band between 3600 and 3100 cm^−1^ (1) corresponded to the OH vibration in MCC. The band between 3000 and 2800 cm^−1^, with the absorption band at 2946 cm^−1^ (2) and the band at 1331 cm^−1^ (4), corresponded to symmetric and asymmetric CH_2_ stretching vibration [46]. However, 1712 cm^−1^ (3) C=O stretching vibrations of the ester group were usually used as one of the key bands to characterize PBS spectra [19,48,49,50]. There was an absorption band for the C-O stretching vibration of PBS at 1150 cm^−1^ (5). The decrease in its intensity reflected a reduction in the crystallinity of the composite material [47]. A shift of this band corresponded to the interaction between the cellulose and polymer chains in the composite [48]. Finally, a band between 1050 and 1010 cm^−1^, with the maximum at 1046 cm^−1^ (6), corresponded to the stretching vibration of the O-C-C. In the APTMS sample’s spectra, a new absorption band was observed at 1557 cm^−1^ and was attributed to the NH scissoring bending vibration [49,50,51]. In the EST sample’s spectra, there were decreased intensities of the ester’s C=O and C-O characteristic bands at 1712 and 1150 cm^−1^, respectively. However, the O-C-C group’s band intensity increased at 1046 cm^−1^. This could indicate the intramolecular interactions between the ester chains and the cellulose surface. An absorption band at 1100 cm^−1^ could be attributed to C-O stretching vibration for the aliphatic ether linkage in the interphase of the cellulose and polymer matrix. In the PHA sample, there was a noticeable increase of the ester’s group bands intensities at 1712 and 1150 cm^−1^, which could have resulted from the formed ester linkages between the cellulose and polymer. In turn, the CDI composition can be characterized with the new bands at 1556 and 1245 cm^−1^, which could correspond to the NH and CN, respectively [52]. The reaction of the carbodiimides and the carboxyl group’s end-groups of the polyester macromolecules could lead to the chain extension, chain crosslinking, and the formation of the urea group linkages. The PMDI spectra has shown absorption bands at 1603 and 1510 cm^−1^, which could be attributed to the C=O urethane group stretching and C=C aromatic rings, respectively; these linkages could have been formed between the isocyanate and the hydroxyl groups on cellulose, and the isocyanate and the carboxylic acid end-groups of the polyester [44,53,54]. The MAH composition was characterized with the characteristic absorption bands at 1712 and 1150 cm^−1^, which could be testament to the formation of the ester linkages between the maleic anhydride and the hydroxyl groups of the cellulose [43].

### 2.3. Thermomechanical Properties

**Figure 5:**
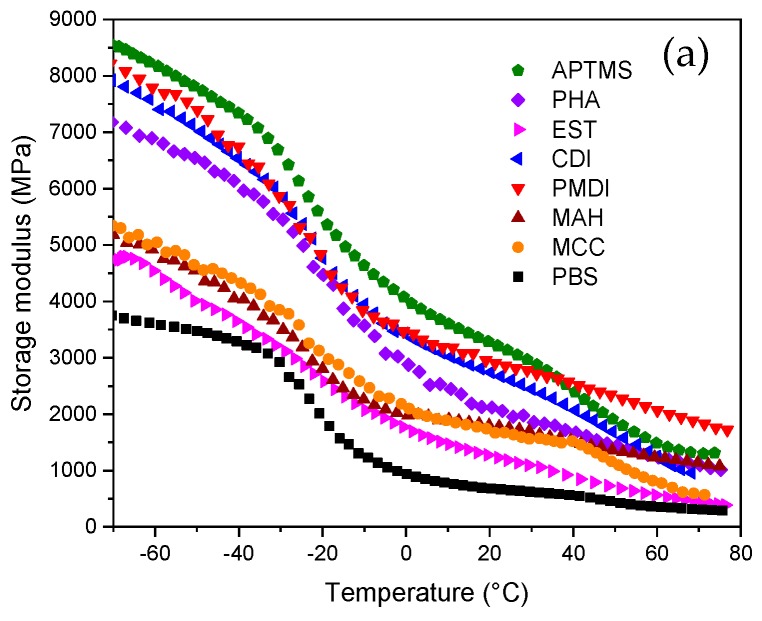

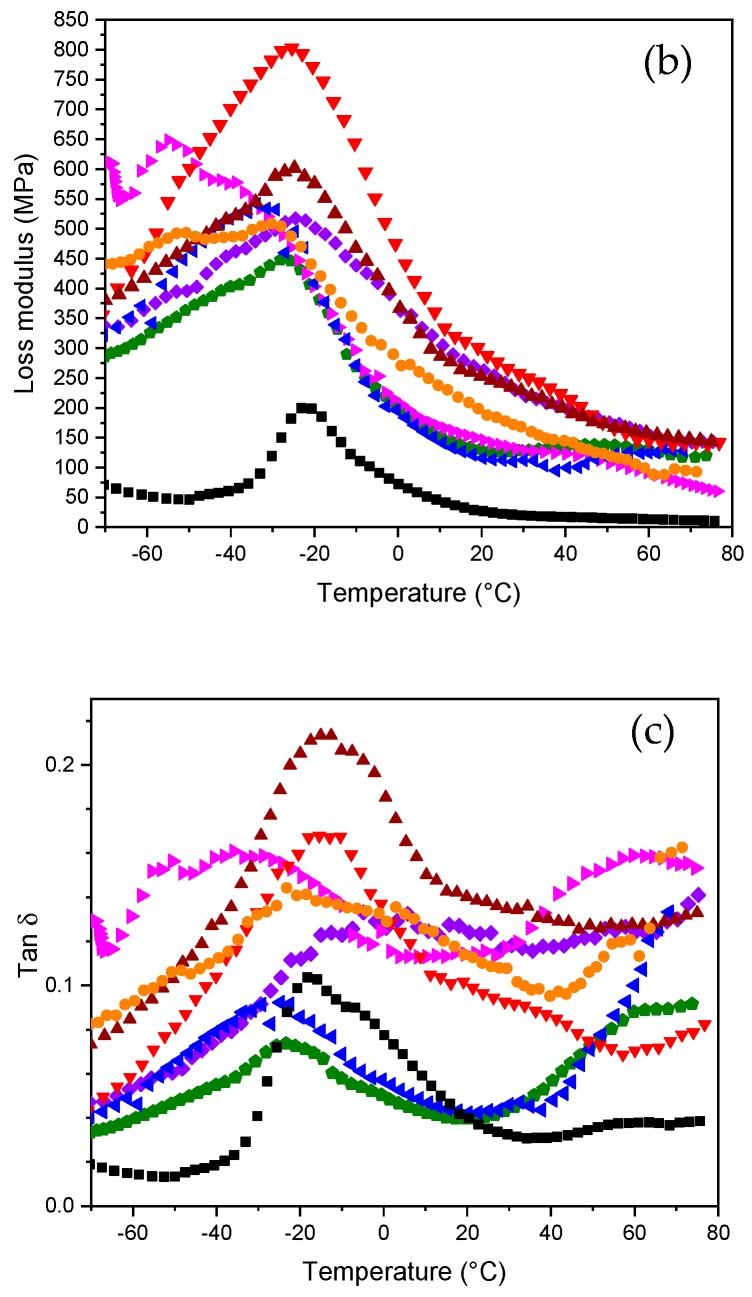
Dynamic mechanical analysis curves of PBS/MCC samples: Storage modulus E′ (**a**), loss modulus E″ (**b**), and loss factor tanδ (**c**).

The dynamic mechanical data of the composites were measured with respect to the temperature (Figure 5). The viscoelasticity of PBS/MCC composite was found to improve and the rigidity dropped by an increase of temperature. Figure 5 showed the temperature dependence of the storage modulus E′, loss modulus E″, and damping factor tan δ for PBS/MCC composites. The PBS had three regions in the DMA curve that could be identified as glassy, glass transition, and rubbery regions. The glass transition region of PBS started with a sharp decrease in storage modulus, which corresponded to the peak in the loss modulus and tan δ graphs. The introduced 70 wt% loading of MCC into PBS polymer could have restricted the overall chain mobility severely, which may have drastically raised its viscosity [55,56]. Accordingly, MCC composites showed less pronounced transitions between the glassy and rubbery states.

MCC acted as a very effective reinforcement and increased the storage and loss modulus strongly, but due to the limited incompatibility of the polymer and matrix, these enhancements could be limited to some extent [10,12,57]. This could be overcome by an increase in crosslinks’ density and enhanced intersegmental interactions for the modified samples, which may have resulted in the higher rigidity of the polymer chains in the composites. This can distribute mechanical stress more evenly in the material and improve mechanical properties [58,59]. The modified compositions are characterized by the improved stiffness after the PHA, CDI, PMDI, and APTMS treatment due to the efficient cross-linking and chain extending of the composite and polymer, respectively [34]. The limited polymer chain flexibility led to a strong increase in material viscoelasticity. Furthermore, the E′ and E″ increased in all temperature ranges for those samples. For example, there was an almost 4-fold increase in E′ for the APTMS composition at room temperature, in comparison to neat PBS. APTMS treatment provided an almost 1.6-fold increase at +70 °C and a 2-fold increase at 0 °C in E′, in comparison to the MCC sample. However, PMDI treatment gave a 2.5-fold increase at +70 °C and 1.7-fold increase at 0 °C. In turn, the EST composition showed pronounced enhancement in viscoelasticity properties and a decrease in stiffness in comparison to MCC, which could be related to plastification of the material by the introduction of flexible long alkyl chains of the ester surfactant molecules [60]. It also showed strong enhancements in E″, which testified to the higher energy that was demanded the polymer viscoelastic deformation [61].

The loss factor tan δ dependence from the temperature is shown in Figure 5c. It corresponded to the efficiency of energy dissipation due to the viscoelastic deformation of the material [27,56]. The representative tan δ varied with temperature and the composition’s nature. MCC did not significantly affect the glass transition temperature of the material, but it did have a significant effect on the magnitude of tan δ absolute values. MCC loading raised the absolute values of the energy dissipation factor significantly because the composite’s viscoelastic deformation required more energy [62]. The noticeable gain in the energy dissipation of the EST composite confirmed the enhancements of the composite’s viscoelastic characteristics due to the long alkyl chains of ester molecules. The characteristic temperatures of the tan δ peak corresponded to a glass transition of the polymer matrix. For example, its value was −15 °C for PBS, −30 °C for EST, and −10 °C for MAH. The shift in the tan δ temperature provided information about the strong enhancement of interactions between polymer chains and filler, and polymer chains due to the enhanced cross-linking, respectively.

### 2.4. Mechanical Properties

**Figure 6:**
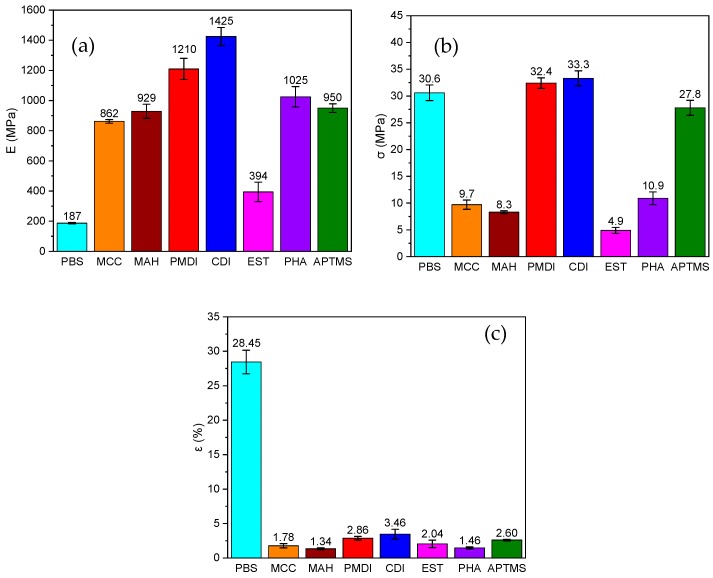
Tensile properties of PBS/MCC composites: (**a**) Elastic modulus E, (**b**) strength σ, and (**c**) strain ε.

The tensile properties of the PBS/MCC composites were tested in the tension mode (Figure S3). The Young’s modulus E increased almost 4.5-fold for the MCC composite, in comparison to neat polymer (Figure 6a). The obtained increase in E can be explained by the reinforcing effect, which is a common characteristic of highly loaded composite materials. As can be observed in Figure 6b,c, there was a significant drop in both tensile strength and tensile strain, as compared to neat PBS. Generally, the addition of wood filler negatively affected the mechanical properties of the composite material. Kajaks et al. reported that the increase of the wood filler amount strongly reduced the strength and ductility of the polyolefin/wood composites [64,65]. For example, for the MCC composite, the obtained E was 862 MPa, the σ was 9.7 MPa, but the ε value was 1.78%. Similar results were also obtained for petrochemical polypropylene (PP) composites [65], which showed an E of 510 MPa, σ of 10.9 MPa, and ε of 5.09%—which was also the case for the PP/wood composite filled with 50% birch plywood sanding dust. Several authors also stressed that the mechanical properties of wood polymer composites are strongly dependent on the filler content, coupling agent, and filler’s modification treatment [66,67,68]. As expected, the additional cross-linking treatment of MCC composites increased the compatibility of the cellulose filler and the polymer matrix. As a result, the E values increased to some extent. The E values of the PMDI, CDI, and APTMS compositions were remarkably higher than for the MCC composite, which means the improvement of compatibility, stress transfer efficiency, and the interfacial strength through the establishment of chemical bonds at the interfaces between cellulose and polymer matrix phases [26]. The E value rose 1.4-fold for PMDI, 1.6-fold for CDI, and 1.1-fold for APTMS, respectively. The tensile strength σ value for those samples were also significantly higher than for the MCC composition and was similar to the initial neat PBS. The obtained σ values and the E values of the modified MCC composites were comparable to the polymer/wood composites reported by several authors [43,64,69].

Generally, all composites showed brittle tensile behavior [35,57], which is also common for high-rigid composites loaded with 70 wt% fillers. A common cause is the formation of aggregates, voids in the composite’s structure, and little bonding between the filler and polymer. This has been clearly evidenced by the obtained SEM micrographs of the fracture surfaces for the processed composites (Figure 3). This brittle behavior can be attributed to the successful chemical modification of MCC during composite preparation, which resulted in cross-linking bonding’s being developed between the filler and polymer. However, MAP, PHA, and EST composites showed moderate improvement in rigidity and a strong decrease in strength and strain, which could have resulted from the poor dispersion and weak bonding between components of the composite material. It was reported that the MCC only interacted strongly with the polymer chains by polar interaction with the chain ester group units [42]. However, the functionalization of the filler surface led to the enhancement of the interaction strength with the developed moieties at the MCC [70]. It must be ascertained whether the chemical bonding of cellulose with the polymer chains through cross-linking contributed to the further enhancement of material stiffness properties or not [57,71]. It should be noted that the remarkable stiffness improvements have generally only been achieved when MCC filler was homogeneously dispersed in the polymer matrix, which could promote strong interfacial interaction and the suppression of polymer chain mobility [72]. Hence, the tendency of particle agglomeration can be diminished by the chemical treatment of its surface [73,74].

## 3. Materials and Methods 

### 3.1. Raw Materials

BioPBS™ FZ71PB^®^ is bio-based and biodegradable polybutylene succinate (PBS) resin, produced by PTT MCC Biochem Company Ltd., to be used for conventional thermoplastic extrusion and injection molding processing and for industrial applications. PBS is characterized by a density of 1.36 g/cm^3^, melt flow index MFI [190 °C, 2.16 kg] 22 g/10 min, and melting point of 115 °C.

Carbodilite^®^ HMV-15CA was purchased from Nisshinbo Chemical Inc. Carbodilite contained a carbodiimide (CDI) group, which worked as a chain extender and hydrolysis stabilizer. Addapt^®^ BioWet 25 L is a solvent-free water-based, readily biodegradable surfactant based on polyhydroxy amides (PHA). Addapt^®^ Ester 80DA is a water and solvent-soluble synthetic aliphatic ester (EST) dispersing agent. BioWet 25 L and Ester 80DA were both purchased from Adapt Chemicals B.V. (3-Aminopropyl)trimethoxysilane (APTMS), maleic acid anhydride (MAH), and acetic acid were purchased from Sigma–Aldrich. Polymeric diphenylmethane diisocyanate IsoPMDI 92140 (PMDI) was purchased from BASF.

### 3.2. Preparation of Microcrystalline Cellulose

**Figure 7:**
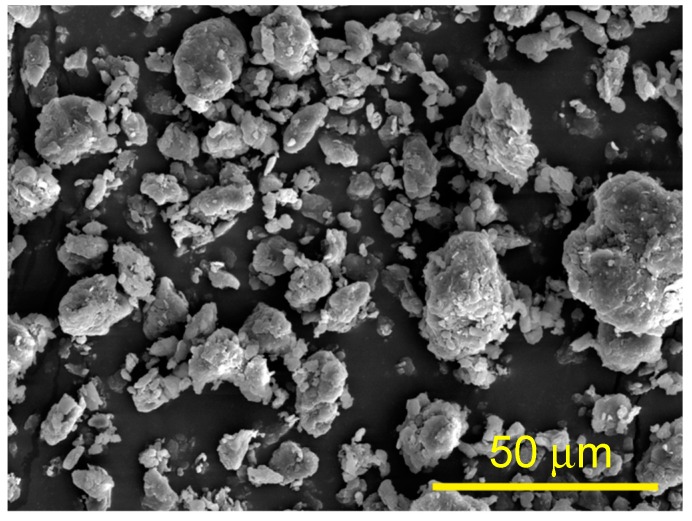
Scanning electron microscopy micrographs of prepared microcrystalline cellulose (MCC).

Microcrystalline cellulose was obtained from TCF bleached softwood kraft pulp (Metsä Botnia AB) according to the procedure reported by the authors [75]. In compliance with this method, the pulp was impregnated with a thermocatalytic degradation catalyst-weak hydrochloric acid solution (0.05%), with a modulus of 1:20. After pressing out the excess liquid, the pulp was thermally treated at a temperature of 120 °C until at a dry state. This facilitated the destruction of the amorphous part of cellulose, while the crystalline one remained almost intact. The degree of polymerization decreased and reached the so-called leveling-off degree of polymerization (LODP), which, in the case of cellulose, was ~250 units [76]. Then, the partially degraded pulp was ground in a ball mill “U.S. Stoneware JAR MILL 755RMV1” (USA) with variable speed. The milling jar was made from alumina-fortified porcelain, with a capacity of 5.7 L. Cylindrical grinding media from corundum 2.1 × 2.1 cm in size were used; the charging factor was 1 kg/L; the grinding time was ~15 h. As a result, microcrystalline cellulose powder was obtained. The microstructure of the obtained microcrystalline cellulose was evaluated by SEM and is shown in Figure 7. It was found that the Zeta potential and average sizes of the obtained MCC particles were about −16.9 mV and 40 μm, respectively.

### 3.3. Processing of PBS/MCC Composites

The aim was to use the high loading of the MCC filler in the composite. To this end, PBS was mixed with 70% (*w*/*w*) cellulose in a thermo-kinetic mixer (Plastograph EC plus 50EHT, Brabender GmbH and Co. KG, Duisburg, Germany). Considering the previous investigations, which showed the possibility of high loadings of cellulose filler for PBS composites manufacturing, the MCC filler content was proposed to be equal to 70 wt%. The processing temperature was set to 130 °C and the screw speed was 70 rpm. In total, 40 g per batch were introduced in the thermo-kinetic mixer for a total mixing time of 7 min. The PBS and MCC were dried in a vacuum chamber at 40 °C for 24 h before composite preparation.

The PBS/MCC composites were ground and compression-molded with a Carver CH 4386 hydraulic press to obtain thin films. The plate temperature was set to 140 °C and the material was preheated for 2 min and was formed with a pressure of 3 metric tons for 3 min, followed by rapid cooling between metal plates at room temperature for 3 min. The dog-bone shape and stripe specimens were cut. These specimens were further tested for tensile, structural, dynamic-mechanical, density, calorimetric, and thermal properties.

### 3.4. Chemical Modification of PBS/MCC Composites

It is well known that the modification of the polymer/cellulose composites is needed to enhance the polymer and cellulose components’ interface compatibility as a means of obtaining high exploitation properties of the final composite material [26]. The strong interfacial adhesion and efficient stress transfer across the phases of the polymer matrix and cellulose filler can be established by different modification additives during the composite processing. Based on this principle, the additives of MAH, PMDI, CDI, EST, PHA, and APTMS were used to adjust the interfacial interactions through compatibilization of the components [77,78,79,80,81,82,83,84]. The used compositions and the modification procedures were selected with regard to the literature data and preliminary tryouts. Altogether, seven different PBS/MCC compositions were obtained by using different chemical modification treatments of composites in order to improve the compatibility between the components (Table 2). The obtained PBS/MCC composites and specimens were stored in sealed bags before any testing.

For PBS/MCC composite modification, 1 wt% of MAH, 1.5 wt% of PMDI, and 3 wt% of CDI were loaded during the melt processing process with the thermo-kinetic mixer [80,81,82,83]. However, modified MCC was blended with PBS without any additional additive loading. The modified MCC preparation was the following: 30 g of MCC was suspended in 500 mL water and the mixture was homogenized with ultrasound sonification for 10 min. Slowly, 90 mL of PHA or EST was added within 2 h; stirring and ultrasound sonification were applied sequentially for 30 min time periods [84]. The acquired modified MCC suspensions were then filtrated and dried in a vacuum. For the silanization, 50 mL of APTMS was dissolved in 250 mL distilled water and stirred, while the solution pH was stabilized to 4 by the addition of acetic acid [51]. 50 g MCC was added to the solution after pH was fixed at 4. The mixture was stirred at room temperature for 2 h, followed by filtration and drying in a vacuum. The chemical reaction and permanent surface modification of MCC was accomplished in a vacuum oven at 120 °C for 2 h.

### 3.5. Testing Methods

Differential scanning calorimetry (DSC) measurements were recorded for previously dried samples in a DSC-1 of Mettler Toledo (USA) under a nitrogen atmosphere. About 10 mg of composite samples were subjected to the whole DSC protocol, in which all the samples were heated to 150 °C at a rate of 10 °C min^−1^, held at that temperature for 5 min, then cooled to 25 °C at a rate of 10 °C min^−1^, followed by 5 min at that temperature and a second heating scan between 25 °C and 150 °C at a rate of 10 °C min^−1^. The crystallization and melting temperatures, enthalpies, and crystallinities, respectively, were calculated from the experimental heating and cooling curves. Melting temperature (T_m_) and the melting enthalpy (ΔH_m_) were measured on the second heating run. Meanwhile, crystallization temperature (T_c_) was obtained on the cooling run.

Thermogravimetric analyses (TGA) were performed on a TGA1/SF apparatus from Mettler Toledo (USA) at a heating rate of 10 °C min^−1^. The thermogravimetric tests were performed on a Mettler TG50 instrument. Specimens of about 10 mg in weight were heated in the air atmosphere from 25–700 °C. The thermal stability of the material was evaluated from the weight-loss heating curves. The weight loss was calculated, according to ASTM D3850, by using the Mettler original software.

Dynamic mechanical analysis (DMA) was carried out on a Mettler DMA/SDTA861e analyzer (USA). The analysis was conducted in tension mode from −70 °C to 80 °C at a 3 °C min^−1^ heating rate and applied the force of 10 N, elongation of 10 μm, and a frequency of 1 Hz. Storage modulus E′, loss modulus E″, and loss factor tan δ = E″/E′ were continuously recorded as a function of the temperature.

Tensile tests were performed at room temperature on a universal testing machine Tinius Olsen model 25ST (USA), equipped with a load cell of 5 kN at 0.2 mm min^−1^ crosshead speed. Dumbbell samples were dried in a vacuum oven for 12 h at 60 °C and were successively pre-conditioned overnight under the environmental conditions of the measurement. Five measurements were performed for each PBS composite at room temperature and ambient conditions.

Density ρ was determined by weighing the material in air and ethanol on Sartorius KB BA 100 electronic scales equipped with a Sartorius YDK 01 hydrostatic density measurement kit. 

FTIR-ATR was used to study the chemical bonding and interactions between the components of the prepared PBS/MCC composites. FTIR-ATR spectra of composites were collected at a resolution of 4 cm^−1^ on a Nicolet 6700 (ThermoScientific, Germany) in the region of 800–4000 cm^−1^. Sixteen measurements of every specimen were performed, and the average spectrum is shown.

Zeta potential and size of particles for MCC were determined on Zeta Sizer Nano ZS90 (Malvern, UK) for 0.05 wt% suspension in distilled water. The MCC particles and composites microstructures were examined with a scanning electron microscope (SEM) Phenom Desktop SEM, at a voltage of 10 kV. The liquid nitrogen fractured surfaces of the composites were used for measurements. 

The dispersion of the MCC in the polymer matrix was examined with an SEM Hitachi Tabletop Microscope TM3000, at a voltage of 15 kV and with several magnifications. The surfaces of the samples were fractured at liquid nitrogen temperatures.

## 4. Conclusions

We report wood-like composites prepared from bio-based polybutylene succinate (PBS) and micro cellulose (MCC) processed from bleached softwood. The prepared PBS/MCC composites are very promising materials as a sustainable woody-like composite alternative to conventional fossil polymer-based wood–plastic composite (WPC) applications. Composites were processed by melt blending and the filler content was selected at 70 wt%. Surface chemical treatment and composition modification were performed to enhance the compatibility between cellulose filler and polymer matrix as well as to enhance several exploitation properties. The structure of the composites was strongly affected by used chemical modification treatment of the MCC used. The chemically modified MCC composites lead to very enhanced mechanical, thermo-mechanical, and thermal properties of the PBS/MCC composites. However, the ductility of the obtained composites strongly decreased after the incorporation of the cellulose; nevertheless, the value of the composites’ appropriate can be suitable to apply this composite material for various construction applications, including profiles, decks, and housing appliances, where high ductility is not compulsory.

## Figures and Tables

**Table 1:** Thermal and physical characteristics of PBS/MCC composites.

Sample	T_m_ (°C)	T_c_ (°C)	H_m_ (J/g)	H_c_ (J/g)	χ_c_ (%)	ρ (g/cm^3^)	*ρ* * (g/cm^3^)
PBS	114.2	75.3	75.1	72.9	68.0	1.365	-
MCC	117.5	79.4	21.4	19.8	64.6	1.370	0.947
MAH	114.7	83.6	16.9	20.4	51.0	1.362	0.925
PMDI	111.3	86.2	15.8	16.0	47.6	1.336	0.851
CDI	113.1	83.4	15.6	16.8	47.1	1.315	0.791
EST	114.8	81.8	19.2	18.2	57.9	1.385	0.990
PHA	115.3	80.0	18.5	20.3	55.8	1.402	1.038
APTMS	115.2	81.3	17.3	20.2	52.2	1.385	0.990

* Calculated by using the MCC density = 1.600 g/cm^3^ [63].

**Table 2:** Obtained PBS/MCC compositions.

Sample	Description
PBS	Neat polymer
MCC	Microcrystalline cellulose
MAH	Maleic acid anhydride
PMDI	Polymeric diphenylmethane diisocyanate
CDI	Carbodiimide
EST	Aliphatic ester
PHA	Polyhydroxyamide
APTMS	(3-Aminopropyl) trimethoxysilane

## Supplementary Materials

The following are available online at https://www.mdpi.com/1420-3049/25/1/121/s1, Figure S1: SEM micrographs of fractured surfaces of PBS/MCC composites; Figure S2: FTIR spectra of PBS/MCC composites; Figure S3: Example of the characteristic tensile curves of PBS/MCC composites.
