# Supplementary material for: Highly Loaded Cellulose/Poly (butylene succinate) Sustainable Composites for Woody-Like Advanced Materials Application
Source: Molecules. 2019 Dec 28;25(1):121. doi: 10.3390/molecules25010121 (PMC6982959; doi:10.3390/molecules25010121)
Supplement: Supplementary file 1 [file molecules-25-00121-s001.pdf]

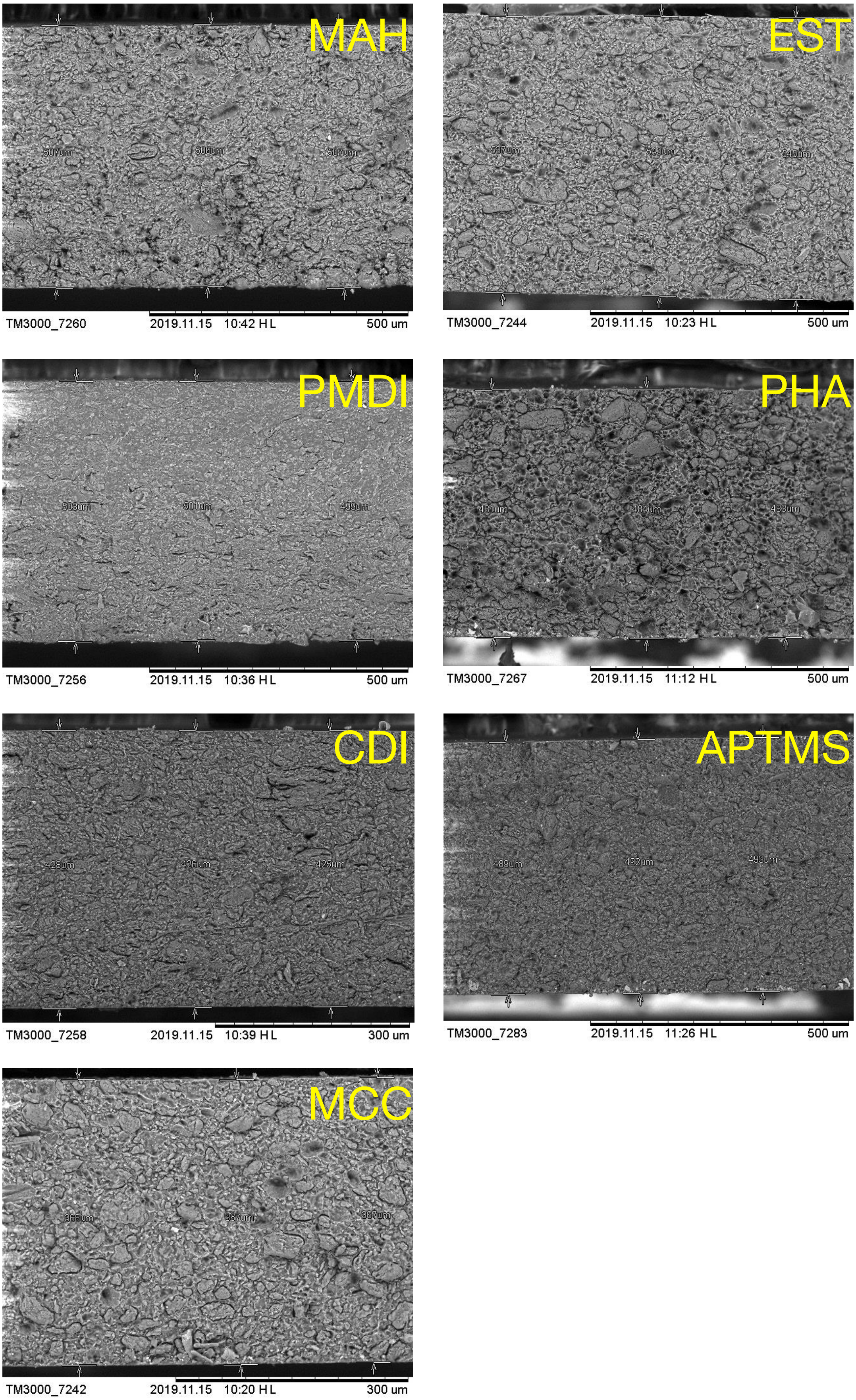

Figure s1. SEM micrographs of fractured surfaces of PBS/MCC composites.

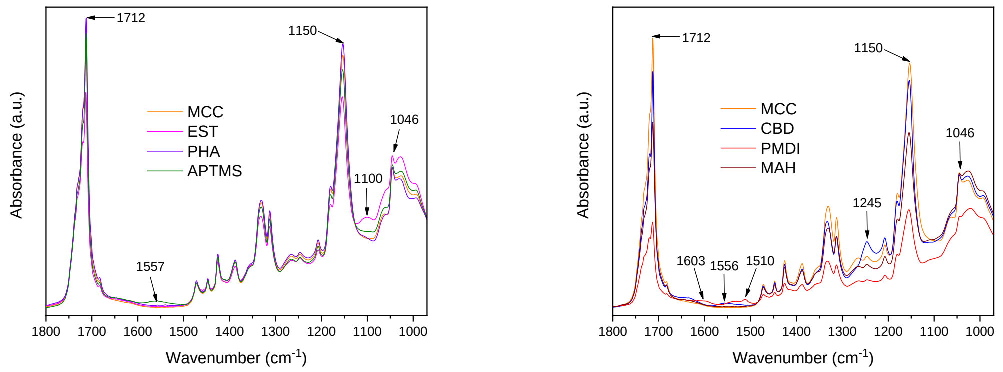

Figure s2. FTIR spectra of PBS/MCC composites.

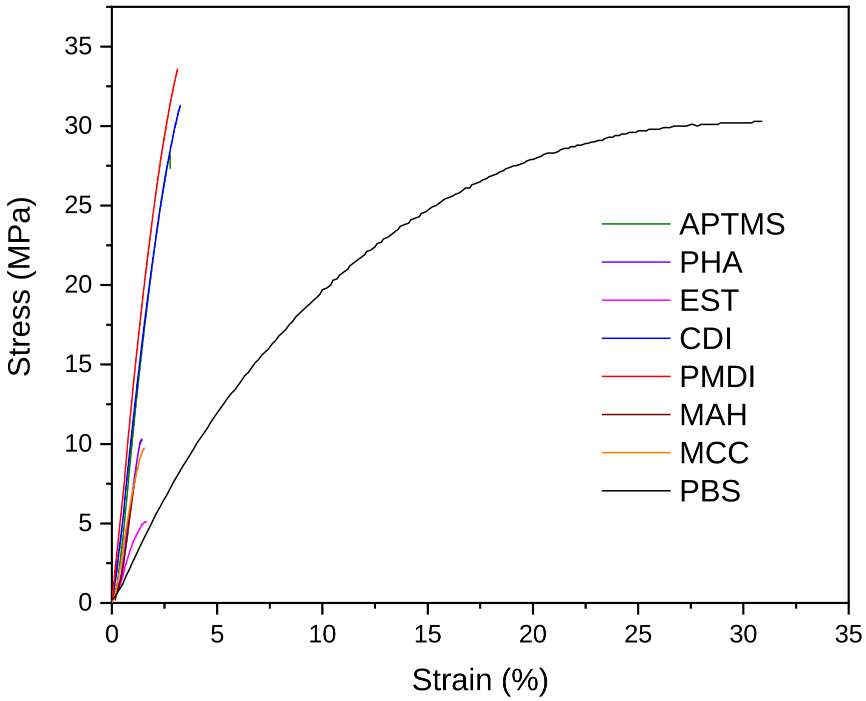

Figure s3. Example of the characteristic tensile curves of PBS/MCC composites.
